# Cage Geometry Reorganizes N···C Contacts While Preserving Nitrogen Spin Localization in N@C_60_ and N@C_70_: A DFT Study

**DOI:** 10.3390/nano16211349

**Published:** 2026-10-24

**Authors:** Tuğba Göcen, Sevgi Haman Bayarı

**Affiliations:** 1 Ahmet Erdoğan Vocational School of Health Services, Zonguldak Bülent Ecevit University, İbn-i Sina Campus, 67600 Zonguldak, Türkiye; 2 Department of Physics Engineering, Faculty of Engineering, Hacettepe University, 06800 Ankara, Türkiye; bayari@hacettepe.edu.tr

**Keywords:** endohedral fullerenes, carbon nanocages, spin localization, density functional theory, noncovalent interaction analysis, QTAIM

## Abstract

Endohedral nitrogen fullerenes are promising candidates for molecular spin qubits and quantum sensing. We compare nitrogen spin localization and N···C contact topology in N@C_60_ and N@C_70_. Baseline structural, vibrational, electronic, population, spin density, noncovalent interaction (NCI), and quantum theory of atoms in molecules (QTAIM) analyses used UB3LYP/6-31G(d). Counterpoise-corrected interaction energies were calculated using UB3LYP-D3(BJ)/def2-TZVP, with def2-QZVP checks. QTAIM sensitivity was assessed at fixed geometries using UB3LYP/def2-TZVP and UωB97X-D/def2-TZVP. Encapsulation leaves both cages essentially unperturbed: selected vibrational shifts are ≤0.8 cm^−1^ and HOMO–LUMO gap changes are ≤0.007 eV. Frontier orbitals remain cage-dominated, whereas Mulliken and Hirshfeld analyses assign 94–99% of the spin population to nitrogen, without substantial charge transfer. Counterpoise-corrected def2-TZVP interaction energies are −9.09 and −8.55 kcal mol^−1^, respectively; def2-QZVP changes each by <0.05 kcal mol^−1^. NCI/QTAIM descriptors indicate weak, low-density N···C contacts. N@C_60_ retains 60 connections across all three tested levels; N@C_70_ has eight at baseline and the same six in both def2-TZVP calculations. Thus, extensive and restricted contact networks remain distinct, although the exact N@C_70_ count and local descriptors depend on computational level. Predominantly nitrogen-centered spin therefore accompanies different surrounding contact topologies in cages that differ in size and shape.

## 1. Introduction

Carbon nanomaterials, from zero-dimensional fullerenes to one-dimensional nanotubes and two-dimensional graphene, offer structurally well-defined platforms whose properties can be tuned at the atomic scale [1,2,3]. Among them, closed carbon nanocages are distinctive because their interior provides a nanoscale confinement space that can host atoms, ions, or small molecules and protect them from the external environment [4,5]. Nitrogen endofullerenes have been proposed for quantum information processing [6]. The broader fullerene literature also covers molecular electronics and biomedical applications [2,4,7].

In endohedral fullerenes, an atom or molecule is confined within a closed sp^2^-carbon cage. The host limits chemical access to the guest. The extent of guest–cage coupling depends on the system [4,5]. Unlike many metallofullerenes, nonmetal endohedral fullerenes are generally not stabilized by extensive charge transfer; their confinement is instead governed largely by weak interactions and steric compatibility [4,5].

C_60_ and C_70_ are well-established fullerene cages with distinct geometrical characteristics [4]. The discovery and subsequent experimental preparation of C_60_ established fullerenes as experimentally accessible carbon nanostructures [8,9]. N@C_60_ and N@C_70_ therefore provide a useful pair for examining the same atomic nitrogen guest in two chemically related but geometrically distinct carbon cavities. Atomic nitrogen is stabilized by encapsulation and largely retains its atomic spin character under ambient conditions [10,11,12].

N@C_60_ was first identified following nitrogen ion implantation by electron paramagnetic resonance (EPR) spectroscopy [10], and its properties were subsequently characterized in greater detail [11]. EPR measurements revealed that nitrogen remains close to the center of the cage and is only weakly coupled to the carbon framework. This behavior has led researchers to describe the fullerene shell as a “chemical Faraday cage” that chemically isolates the nitrogen atom while largely preserving its atomic character [12]. EPR results also show that hyperfine coupling and g values differ slightly for N@C_70_ compared to those of N@C_60_ [12].

Experiments show that nitrogen can retain its atomic spin character while remaining sensitive to the host and its surroundings. Molecular alignment and EPR measurements reveal that spin behavior depends on the environment [13,14]. In a direct comparison in liquid CS_2_, Morton et al. resolved an additional relaxation contribution in N@C_70_ associated with reorientation of its permanent zero-field splitting [15]. Hui et al. later incorporated N@C_60_ into MOF-177 and detected environmental protons by electron spin echo envelope modulation [16]. These observations motivate comparing the spatial N···C contact patterns in the two carbon cavities.

Kobayashi et al. used B3LYP and MP2 calculations to compare the electron spin density at the nitrogen nucleus and isotropic hyperfine coupling constants in N@C_60_ and N@C_70_ [17]. Both quantities were slightly larger in N@C_60_, which was attributed to its smaller cavity. The vibrational properties of C_60_ and C_70_ have been studied experimentally and computationally [18,19,20,21,22] and the response of N@C_60_ to an external electric field has also been examined [23]. Reduced density gradient, charge transfer, orbital interaction and energy decomposition analyses have been applied to other endohedral C_60_ systems [24]. EPR and hyperfine measurements sensitively probe the local spin environment, but do not show how the weak interaction is distributed in the total electron density. Interaction energies for nonmetal endohedral fullerenes must also be interpreted with attention to basis set superposition error and thermodynamic context [5].

Computational studies of nonmetal guests encapsulated in fullerenes indicate that cage size and shape influence confinement energetics and can modify guest orientation or geometry [5,25,26,27]. Bil, Hutter, and Morrison also reported little charge transfer and spin localized on nitrogen in their ab initio molecular dynamics study of N@C_70_ [28].

Related studies have examined fullerene assemblies and reactions involving more than one guest under confinement. Bandyopadhyay and Berakdar studied spin interactions mediated by fullerene orbitals in assemblies of fullerenes containing nitrogen [29]. Nunes Rodrigues et al. combined Car–Parrinello dynamics with QTAIM, reduced density gradient, and interaction region indicator analyses to examine how N–H_2_ reactivity under C_70_ confinement depends on spin [30]. These studies provide context for our comparison of neutral quartet N@C_60_ and N@C_70_, each containing a single guest, at fixed optimized geometries.

Earlier calculations described the atomic electronic structure of N@C_60_ and compared the electron spin densities at the nitrogen nuclei and hyperfine couplings of N@C_60_ and N@C_70_ [17,31]. Here, we examine what the spatial distribution of the calculated total electron density adds to the established picture of spin localized on nitrogen. We compare N···C paths between individual atom pairs with the baseline spin populations and separately calculated interaction energies. We also examine which features of the contact graph persist when the density model changes. Retention of nitrogen atomic character provides the reference for this comparison and is not presented as a new finding.

C_60_ and C_70_ enclose the same neutral quartet nitrogen guest in cages made entirely of carbon, but differ in both size and shape. Their comparison probes the overall confinement geometry; it does not isolate shape at a fixed cage size. We examine whether similar nitrogen spin localization occurs with similar or different N···C contact patterns. Bond path counts are not treated as measures of bond strength.

The present study tests the hypothesis that changing the host from nearly spherical C_60_ to elongated C_70_ primarily reorganizes the spatial distribution of weak N···C contacts while leaving the cage structure and nitrogen-centered spin largely intact. C_60_, C_70_, N@C_60_, and N@C_70_ were therefore analyzed using geometry optimization, harmonic frequencies, frontier orbitals, total and projected densities of states, atomic charges, spin populations, spin density, counterpoise-corrected interaction energies, NCI, and QTAIM. At the baseline level, the host structure and spin localized on nitrogen are largely preserved, while the spatial contact graph changes. Checks of search convergence and sensitivity to the basis set and functional are reported separately from this baseline comparison.

## 2. Materials and Methods

### 2.1. Electronic Structure Calculations and Vibrational Analysis

Electronic structure calculations were carried out with Gaussian 16, Revision C.01 [32]. All four systems were considered in the gas phase. Neutral C_60_ and C_70_ were described as closed-shell singlets at the B3LYP/6-31G(d) level. N@C_60_ and N@C_70_ were described as open-shell quartets using unrestricted UB3LYP/6-31G(d) [17,33,34,35]. Geometry optimizations of the endofullerenes were conducted without symmetry constraints. GaussView 6.0 was used to inspect the optimized structures and frontier molecular orbitals [36].

For each system, harmonic frequencies were calculated at the same level of theory as the corresponding geometry optimization. No imaginary frequencies were found; the optimized structures therefore correspond to local minima. Infrared (IR) intensities and Raman activities were obtained from the same calculations. The final frequency calculations used an ultrafine integration grid and tight SCF convergence criteria.

The 6-31G(d) basis provided a common polarized double-zeta reference for comparing geometries and harmonic frequencies. Kobayashi et al. also used UB3LYP/6-31G(d) to optimize nitrogen endofullerene geometries [17]. This precedent supports the choice of the baseline structural protocol. It does not establish convergence of the weak interaction energies or electron density topology.

The vibrational frequencies are reported as unscaled harmonic values. No empirical scaling factor was applied. The empty cages and nitrogen endofullerenes were compared at the same level of theory. The unscaled values were not treated as direct quantitative predictions of experimental band positions. Selected vibrational modes are discussed in the main text. The complete frequencies, infrared intensities, and Raman activities are provided in the S2_Vibrational_Data worksheet of the Supplementary Data workbook.

### 2.2. Interaction Energy Calculations

The interaction energy between the N atom and the fullerene cage was estimated by treating N and C*_n_*, where *n* = 60 or 70, as separate fragments. Basis set superposition error was corrected with the counterpoise method introduced by Boys and Bernardi [37]. The corrected interaction energy was obtained from

ΔE_int,CP_ = E(N@C_n_) − E_CP_(C_n_) − E_CP_(N) (1)

In this equation, E(N@C_n_) is the total energy of the endofullerene. E_CP_(C_n_) is the counterpoise energy of the cage fragment, and E_CP_(N)is that of the nitrogen fragment. The fragment energies were calculated at the fixed geometry of the endofullerene.

Counterpoise calculations were carried out at the UB3LYP/6-31G(d) level. A second set used the UB3LYP-D3(BJ)/def2-TZVP level [38,39,40]. At both levels, the quartet geometries optimized at UB3LYP/6-31G(d) were used without further geometry optimization. The reported values refer to electronic interaction energies. Additional counterpoise single-point calculations at UB3LYP-D3(BJ)/def2-QZVP used exactly the same nuclear coordinates, fragment assignments, and reference states as the def2-TZVP calculations. The complete endofullerenes and isolated nitrogen fragments were neutral quartets, and the cage fragments were neutral singlets.

For noncovalent interaction energies, B3LYP-D3(BJ)/def2-QZVP with full counterpoise correction was reported to give an RMS deviation of 0.23 kcal mol^−1^ from the revised S66×8 reference data based on coupled-cluster calculations [41]. This benchmark supports the choice of method for the interaction energies, but does not establish its error for the present open-shell endofullerenes.

The additional interaction energy calculations used def2-TZVP, a larger polarized triple-zeta valence basis in the Karlsruhe def2 family [40]. This family allows for comparison of def2-TZVP with def2-QZVP while keeping the functional and dispersion treatment unchanged. The nuclear geometries were also held fixed so that changes in the electronic treatment were assessed without geometry relaxation. Changing the basis set family alone does not establish improved accuracy. The comparison of UB3LYP/6-31G(d) with UB3LYP-D3(BJ)/def2-TZVP changes both the basis set and dispersion treatment, so it does not isolate basis set convergence. The separate UB3LYP comparison at fixed geometries in Section 2.4 and Section 3.9 examines the N@C_60_ and N@C_70_ density topologies.

The comparison of def2-TZVP with def2-QZVP used the same functional, D3(BJ) correction, UltraFine integration grid, and SCF = (XQC,Tight, MaxCycle = 512) settings. The Gaussian cutoff for overlap eigenvalues was 10^−6^ at both basis sizes. At def2-QZVP, the calculations for the complete systems retained 3465 of 3477 basis combinations for N@C_60_ and 4031 of 4047 for N@C_70_. This comparison tests how the electronic interaction energies at fixed geometries depend on basis size under the same numerical settings. No additional geometry optimization or frequency calculation was performed at def2-QZVP.

In the counterpoise procedure, the electronic energies of the fragments were evaluated in the full endofullerene basis, with ghost functions on the other fragment and no relaxation of either fragment. For the chosen damping parameters, the additive D3(BJ) correction depends on the real atoms and their fixed nuclear geometry. Ghost centers do not introduce atoms into the dispersion correction [38,39]. In all four counterpoise jobs, each fragment had the same D3(BJ) term in the full endofullerene basis and in its own basis. These terms therefore cancel from the BSSE correction. The D3(BJ) term of the complex minus those of the fragments is already included in the reported counterpoise interaction energies and was not added again.

### 2.3. Population, Frontier-Orbital, and Density-of-States Analyses

Atomic charges were obtained from Mulliken and Hirshfeld population analyses, and CM5 charges were also calculated [42,43]. Mulliken and Hirshfeld spin populations were used to estimate the fraction of the spin population located on nitrogen. For each method, the nitrogen spin fraction was defined as the nitrogen spin population divided by the sum of the atomic spin populations over all atoms.

HOMO and LUMO energies were taken from the converged wavefunctions of the optimized structures. The energy gap was calculated as

ΔE = E_LUMO_ − E_HOMO_ (2)

For C_60_ and C_70_, one gap value was obtained for each closed-shell system. For N@C_60_ and N@C_70_, the alpha and beta spin channels were treated separately. These gap values were used only to compare the systems. They were not treated as optical excitation energies or experimental optical gaps.

Total density of states (TDOS) and fragment-projected density of states (PDOS) analyses were carried out with Multiwfn version 2026.1.12 [44,45]. Each discrete molecular orbital energy was broadened with a Gaussian function. A full width at half maximum of 0.30 eV was used over the energy range from −25 to +5 eV. Mulliken partitioning was used to separate the contributions of the carbon cage and nitrogen, which were defined as different fragments. For N@C_60_ and N@C_70_, the alpha and beta TDOS and PDOS data were exported separately. In the figures, the beta curves were reflected below the horizontal axis to make comparison with the alpha curves easier. Values shown below the horizontal axis were used only for display and do not indicate a negative density of states.

### 2.4. Spin Density, NCI, and QTAIM Analyses

The spin density was defined as

*ρ_spin_*(***r***) = *ρ_α_*(***r***) − *ρ_β_*(***r***) (3)

where *ρ*_α_ and *ρ*_β_ are the alpha and beta electron densities, respectively. Spin density grids were obtained from the SCF densities in the UB3LYP/6-31G(d) formatted checkpoint files and visualized with GaussView 6.0. The positive spin density was plotted at the same isovalue of +4.0 × 10^−4^ a.u. for N@C_60_ and N@C_70_. Additional views at +2.0 × 10^−5^ and −2.0 × 10^−5^ a.u. were prepared for both systems (Figure S1). The positive and negative surfaces were shown separately and together, with the carbon cages displayed as wireframes. Blue denotes positive spin density and green denotes negative spin density. The lower threshold was used to show weak regions of both signs. It does not change the underlying electronic calculation.

The NCI and baseline QTAIM analyses of N@C_60_ and N@C_70_ used the same Gaussian formatted checkpoint (.fchk) files, which contain the UB3LYP/6-31G(d) wavefunctions at the corresponding optimized geometries. These analyses used Multiwfn version 2026.1.12 [44,45]. The UB3LYP-D3(BJ)/def2-TZVP and def2-QZVP counterpoise interaction energy calculations described in Section 2.2 were carried out separately.

Noncovalent interaction analysis was based on the reduced density gradient, RDG, and the sign(*λ*_2_)*ρ* functions calculated from the DFT wavefunctions with Multiwfn [44,45,46]. Here, *λ*_2_ is the second eigenvalue of the Hessian matrix of the electron density. The cube files were viewed with VMD [47]. For all four systems, the NCI surfaces were shown at an RDG isovalue of 0.5 and colored with sign(*λ*_2_)*ρ* values over the common range from −0.04 to +0.04 a.u.

For N@C_60_ and N@C_70_, the RDG and sign(λ_2_)ρ cube files used Cartesian grids of 121 × 119 × 123 and 129 × 116 × 118 points, respectively. The spacing along each axis was 0.133174 bohr for N@C_60_ and 0.140221 bohr for N@C_70_. The two cube files for each system used matching grids. In the archived cube data, points with |sign(λ_2_)ρ| ≥ 0.05 a.u. had RDG values set to 100, consistent with masking high-density regions at a threshold of 0.05 a.u. This inspection documents the archived datasets; it does not test grid convergence.

Quantum theory of atoms in molecules (QTAIM) analyses of N@C_60_ and N@C_70_ were carried out with Multiwfn [44,45,48]. Critical points were searched across the molecular electron density, and the topological consistency of the set was checked with the Poincaré–Hopf relation. Satisfying this relation was not considered proof that every critical point had been found. N···C topological paths linked to the relevant (3, −1) critical points were then generated. For each N···C (3, −1) critical point, the electron density ρ*_b_*, the Laplacian of the electron density ∇^2^ρ*_b_*, the kinetic energy density *G_b_*, the potential energy density *V_b_*, the total energy density *H_b_* = *G_b_* + *V_b_* and the ratio *G_b_*/∣*V_b_*∣ were evaluated. The electron localization function (ELF) and sign(*λ*_2_)*ρ_b_* were also calculated at the same critical points [48,49]. Selected individual baseline descriptors are provided in the S3_QTAIM_Data worksheet of the Supplementary Data workbook. Additional descriptors, including G and V, are listed in the Raw_NC worksheet of the QTAIM Sensitivity Data workbook. The local total energy density, H = G + V, was interpreted using the framework of Cremer and Kraka [50].

The documented baseline searches used the Newton algorithm with a maximum of 120 iterations, a convergence threshold of 10^−6^ a.u. for the gradient norm, and a convergence threshold of 10^−7^ bohr for displacement. Starting points were generated from nuclear positions, midpoints between atom pairs, centers of triangles formed by three atoms, and centers of tetrahedra formed by four atoms. The step scale factor was 1.0, the minimum distance used to distinguish critical points was 0.03 bohr, and the screening distance between atoms was 1.50 times the sum of their van der Waals radii. The Hessian singularity criterion was 5 × 10^−22^, and all critical point types were retained. Paths were generated with a step size of 0.03 bohr, a maximum of 451 points per path, and a stopping distance of 0.05 bohr from a critical point.

To test sensitivity to the convergence criteria for critical point searches, the searches were repeated from scratch using the same baseline wavefunctions and fixed geometries. The maximum number of iterations was increased to 200, the threshold for the gradient norm was tightened to 10^−7^ a.u., and the displacement threshold was tightened to 10^−8^ bohr. All other search and path generation settings were unchanged. This test examined numerical search settings separately from sensitivity to the basis set or exchange–correlation functional.

Four additional wavefunctions were calculated for N@C_60_ and N@C_70_ at UB3LYP/def2-TZVP and UωB97X-D/def2-TZVP [51], using the unchanged geometries optimized at UB3LYP/6-31G(d). The Gaussian calculations used an ultrafine integration grid, tight SCF convergence, and an electronic stability analysis (Stable = Opt). Each solution was stable under the perturbations considered. A subsequent single-point calculation read that solution from its checkpoint. The resulting formatted checkpoint files were analyzed with Multiwfn 2026.1.12 using the tighter search protocol described above. Bond critical points were matched by the atom pairs they connect, rather than by their numerical indices. The N@C_70_ descriptor comparisons used the six contacts present at all three levels; contacts that were not recovered were not assigned zero values. These density comparisons kept the geometries fixed. They do not represent def2-TZVP geometry optimizations or benchmarks at the complete basis set limit. The electronic calculation details and limited targeted search for N@C_70_ are documented in Table S6 and the accompanying notes.

## 3. Results

### 3.1. Optimized Structures and Cage Deformation

**Figure 1:**
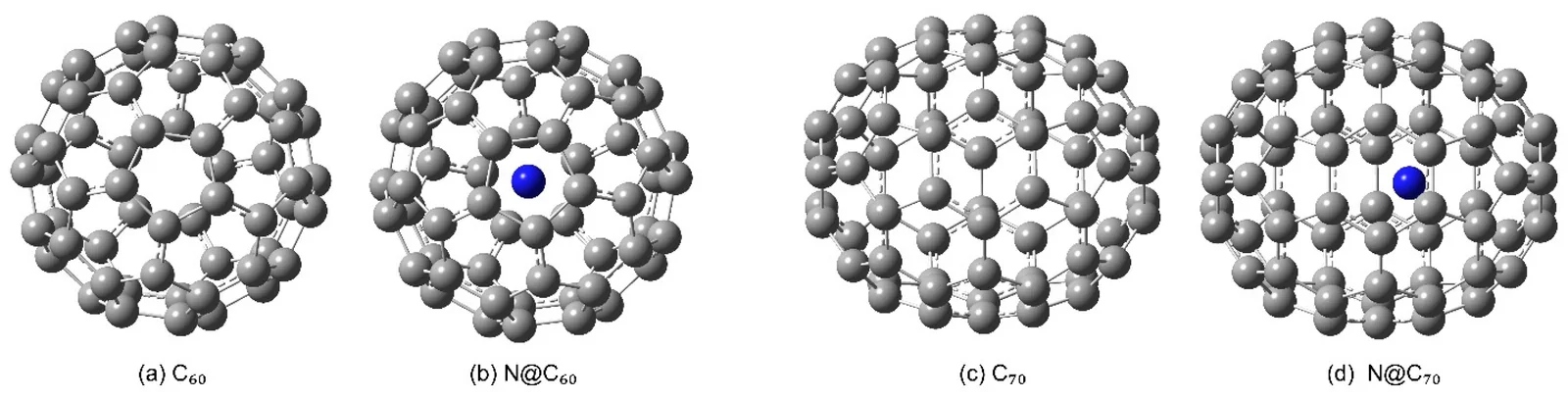
Optimized structures of (**a**) C_60_, (**b**) N@C_60_, (**c**) C_70_ and (**d**) N@C_70._

The optimized geometries retain the characteristic nearly spherical structure of C_60_ and the elongated structure of C_70_ (Figure 1). The optimized Cartesian coordinates of all four systems are provided in the S1_Coordinates worksheet of the Supplementary Data workbook. The geometric center of each cage was calculated as the arithmetic mean of the Cartesian coordinates of all carbon atoms, and the cage radius was defined as the distance from this center to each carbon atom. In N@C_60_, the N atom is essentially centered, with a displacement of 0.001 Å from the geometric center; in N@C_70_, it remains close to the center, with a displacement of 0.017 Å. After optimal rigid alignment, the carbon framework RMSD between each endofullerene and the corresponding empty cage is below 0.001 Å. The mean C–C bond lengths are unchanged to the reported precision, and the bond length ranges differ only marginally (Table 1). Thus, nitrogen encapsulation does not produce a chemically meaningful reconstruction of either cage.

The main structural difference between the two endofullerenes originates from the geometry of the cavity. In N@C_60_, the N···C distances are narrowly distributed between 3.549 and 3.551 Å, consistent with the high symmetry of the nearly spherical cage. In contrast, N@C_70_ exhibits a broader N···C distance distribution of 3.548–4.161 Å because the elongated cage provides inequivalent carbon environments. This wider range reflects the intrinsic anisotropy of C_70_ rather than additional deformation induced by encapsulation. These results are consistent with previous theoretical and EPR studies showing that the encapsulated N atom remains close to the cage center and retains largely atomic character with only weak perturbation of the fullerene host [12,17].

Taken together, the sub-picometer carbon-framework RMSDs and unchanged mean C–C bond lengths show that nitrogen encapsulation preserves the parent nanocage geometry rather than reconstructing the carbon framework; the main structural difference lies in the size and shape of the parent cavities.

### 3.2. Harmonic Vibrational Analysis and Selected IR and Raman Features

No imaginary frequencies were found for any of the four optimized structures, which is consistent with their assignment as local minima on the potential energy surface. The lowest calculated wavenumbers for the empty C_60_ and C_70_ cages are 265.9 and 224.1 cm^−1^, respectively. For N@C_60_, three additional low-frequency modes occur at 66.2, 70.2, and 73.1 cm^−1^. The corresponding modes for N@C_70_ occur at 67.9, 68.0, and 73.4 cm^−1^. Inspection of the normal mode displacements suggests that these vibrations mainly involve motion of the nitrogen atom relative to the cage. This assignment agrees with earlier theoretical and spectroscopic descriptions of N@C_60_, in which a low-frequency mode was assigned to motion of the N atom around the cage center [52].

Table 2 contains a small set of representative cage modes from the lower and higher wavenumber regions of the IR and Raman results. The calculated IR intensities and Raman activities of the additional low-frequency N-dominated modes are very small.

Empty cage and endofullerene modes were matched using the similarity of these patterns rather than wavenumber proximity alone. Closely spaced components were considered as a mode group when a separate assignment was not clear. The complete unscaled results are given in the S2_Vibrational_Data worksheet of the Supplementary Data workbook. After nitrogen encapsulation, the selected wavenumbers of cage vibrations change little, with a largest absolute shift of 0.8 cm^−1^. This observation concerns the selected wavenumbers and does not imply that all IR intensities or Raman activities remain unchanged. This result agrees with the small structural differences described in Section 3.1 and suggests that the N atom produces only a limited change in the carbon frameworks.

Several closely spaced Raman-active cage modes occur between 1605 and 1606 cm^−1^ for both C_70_ and N@C_70_. A reliable match between the individual modes could not be made in this region, so their Raman activities were not compared separately in Table 2.

### 3.3. Interaction Energies, BSSE, and Dispersion Effects

Table 3 lists the counterpoise-corrected interaction energies and the basis set superposition error (BSSE) values for N@C_60_ and N@C_70_. At the UB3LYP/6-31G(d) level, the corrected interaction energies are +3.77 and +2.52 kcal mol^−1^, respectively. Since both values are positive, the results at this level do not suggest net electronic stabilization for either system. The BSSE values are 2.56 kcal mol^−1^ for N@C_60_ and 2.26 kcal mol^−1^ for N@C_70_. These values indicate that basis set superposition can noticeably affect the interaction energies obtained with the 6-31G(d) basis set.

At the UB3LYP-D3(BJ)/def2-TZVP level, the corrected interaction energies are −9.09 kcal mol^−1^ for N@C_60_ and −8.55 kcal mol^−1^ for N@C_70_. The corresponding BSSE values are lower, 0.48 and 0.41 kcal mol^−1^, respectively. We used the additive D3(BJ) terms in the Gaussian counterpoise outputs to calculate the difference between the empirical dispersion correction for each complete endofullerene and the sum of the corrections for its fragments. The resulting values are about −12.17 kcal mol^−1^ for N@C_60_ and −10.27 kcal mol^−1^ for N@C_70_. They were calculated by subtracting the sum of the fragment D3(BJ) terms from the D3(BJ) term of the complete endofullerene. The reported SCF and counterpoise energies already include the D3(BJ) correction, so it was not added again. Removing the difference in D3(BJ) corrections gives UB3LYP/def2-TZVP interaction components of about +3.08 and +1.71 kcal mol^−1^, respectively. Their positive signs are consistent with the pattern obtained at the UB3LYP/6-31G(d) level. Within the adopted model, the empirical D3(BJ) correction changes these positive interaction components into negative total interaction energies. This procedure does not quantify the electrostatic, exchange, and induction terms separately and is not a SAPT calculation or an independent energy decomposition analysis.

At def2-QZVP, counterpoise correction gives interaction energies of −9.13 kcal mol^−1^ for N@C_60_ and −8.52 kcal mol^−1^ for N@C_70_, with BSSE values of 0.12 and 0.16 kcal mol^−1^, respectively (Table 3). Using the unrounded energies, the changes relative to def2-TZVP are −0.04009 and +0.03302 kcal mol^−1^. The interaction energies at fixed geometries therefore change little with this basis enlargement within UB3LYP-D3(BJ). This comparison of finite basis sets does not establish the complete basis set limit or independently validate the functional.

The D3(BJ) correction differences are the same at def2-TZVP and def2-QZVP because the nuclear geometries and dispersion parameters are unchanged. At def2-QZVP, subtracting these differences leaves underlying UB3LYP interaction components of approximately +3.04 kcal mol^−1^ for N@C_60_ and +1.75 kcal mol^−1^ for N@C_70_. The empirical dispersion correction therefore still produces net attraction with the larger basis.

After counterpoise correction, the interaction energies differ by approximately 0.54 kcal mol^−1^ at def2-TZVP and 0.61 kcal mol^−1^ at def2-QZVP. N@C_60_ has a slightly more attractive interaction in both calculations, while the estimated electronic stabilization remains comparable for the two cages. The difference alone does not seem sufficient to support a clear energetic preference for either cage. The reported quantities are fixed-geometry electronic interaction energies. They should not be treated as thermodynamic free energies or conventional bond dissociation energies. The spatial character of the weak interaction between nitrogen and each cage is considered further using the NCI and QTAIM results.

### 3.4. Charge Distribution and Spin Localization

Table 4 lists the atomic charge assigned to nitrogen by three population methods. The Mulliken charges are close to zero, with −0.001 e for N@C_60_ and −0.004 e for N@C_70_. Hirshfeld analysis gives +0.111 and +0.083 e, while CM5 gives +0.092 and +0.071 e. The values vary with the method, but none of the three sets suggests a large ionic charge transfer between nitrogen and the cage.

The small nitrogen charges are consistent with earlier descriptions of limited charge transfer in N@C_60_. Greer reported a Mulliken electron population of 6.982 at the ROHF/DZP level, equivalent to a nitrogen charge of +0.018 e [31]. Manz’s PBE/def2-TZVPP benchmark gave Hirshfeld and CM5 charges of +0.139 and +0.120 e, respectively [53], compared with +0.111 and +0.092 e here. For N@C_70_, Bil et al. reported an average Mulliken nitrogen charge of −0.05 ± 0.03 e over their trajectory at 298 K [28], also indicating limited redistribution. These comparisons are qualitative: the electronic structure methods differ, and the molecular dynamics result includes sampling at finite temperature. Our Mulliken charges are slightly negative, whereas the Hirshfeld and CM5 charges are positive. Neither the direction nor the exact magnitude of this small redistribution should therefore be regarded as independent of the population scheme.

Mulliken and Hirshfeld analyses both assign most of the spin population to nitrogen. The Mulliken values are 2.972 for N@C_60_ and 2.978 for N@C_70_, corresponding to 99.1% and 99.3% of the total spin population, respectively. The Hirshfeld values are 2.823 and 2.865, corresponding to 94.1% and 95.5%. The percentages differ between the methods. In both cases, most of the spin population is assigned to nitrogen.

Spin localization on nitrogen is consistent with the atomlike S = 3/2 state identified by EPR for N@C_60_ [10]. An atomic spin population and the contact spin density at a nucleus are different quantities. Kobayashi et al. optimized the fullerene geometries at UB3LYP/6-31G(d) and evaluated the spin properties with several approaches, including MP2 with the uc-Huzinaga+(2df) basis on nitrogen and 6-31G on the cage carbons [17]. They obtained isotropic hyperfine couplings of 14.75 MHz for N@C_60_ and 13.73 MHz for N@C_70_, reflecting the larger contact spin density in N@C_60_. Our slightly larger atomic spin populations on nitrogen in N@C_70_ describe a different quantity and do not contradict that ordering.

Nitrogen was also reported to retain its atomic character in N@C_60_ using B3LYP/6-311G* and B3LYP-GD2/6-311G* [54]. Wittmann et al. measured an electron spin g factor of 2.00204(4) for N@C_60_ dispersed in solid C_60_ [55]. The measured g factor provides experimental context for the nitrogen spin environment, but it does not determine the nitrogen spin fractions in Table 4. The 94–99% range reflects the assignments from the population schemes; it is not an experimental confidence interval.

The calculated dipole moment is below 0.001 D for N@C_60_ and 0.001 D for N@C_70_. These small values are compatible with the position of nitrogen near the cage center and suggest little charge separation.

### 3.5. Frontier Molecular Orbitals

**Figure 2:**
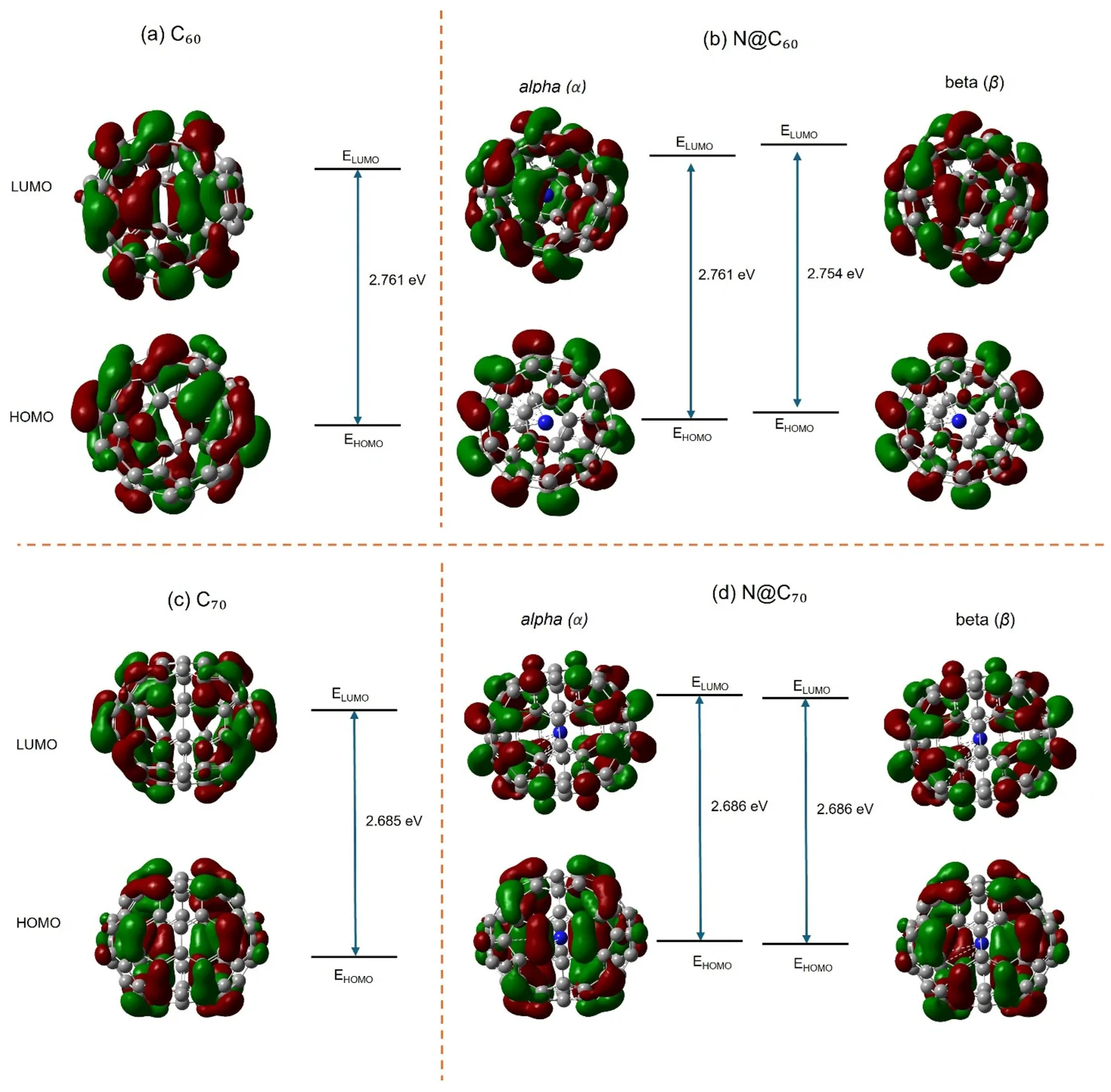
HOMO and LUMO isosurfaces and orbital energy gaps of C_60_, N@C_60_, C_70_, and N@C_70._

The HOMO and LUMO isosurfaces are located mainly on the carbon cages in all four systems (Figure 2). The energy gap between the HOMO and LUMO is 2.761 eV for C_60_. For N@C_60_, the corresponding values are 2.761 eV for the α channel and 2.754 eV for the β channel. C_70_ has a smaller gap of 2.685 eV. In N@C_70_, both spin channels give 2.686 eV at the reported precision (Table 5). The gaps were calculated from the unrounded orbital energies. Table 5 gives the rounded orbital energies and gap values.

The changes in frontier orbital spacing after nitrogen encapsulation are small. The largest absolute difference from the corresponding empty cage is 0.007 eV for the β channel of N@C_60_. By comparison, the gap of empty C_70_ is 0.075 eV smaller than that of empty C_60_. At the present level of theory, the difference between the C_60_ and C_70_ cages appears to affect the frontier orbital spacing more than nitrogen encapsulation.

The frontier orbitals lie mainly on the cage, consistent with Greer’s finding that the occupied 2p states derived from nitrogen lie below the N@C_60_ HOMO and that the cage HOMO changes by no more than 0.008 eV upon encapsulation [31]. This is a shift in the HOMO energy, not a change in the HOMO–LUMO gap, and therefore cannot be equated with the maximum gap change of 0.007 eV found here. Jakes et al. found the UV/Vis spectrum of N@C_60_ to be indistinguishable from that of C_60_ within experimental error [56]. This supports a small perturbation of the cage electronic structure, although the optical spectrum is not a direct numerical benchmark for the orbital gaps calculated for the ground state in Table 5.

The orbital isosurfaces for the α and β channels of the open-shell systems show slight differences, but the frontier orbitals remain located mainly on the carbon cages. The nitrogen contribution to the electronic structure is considered more directly in the projected density of states and spin density analyses. The values in Table 5 are ground-state orbital energy differences used only to compare the systems.

### 3.6. Total and Projected Density of States

**Figure 3:**
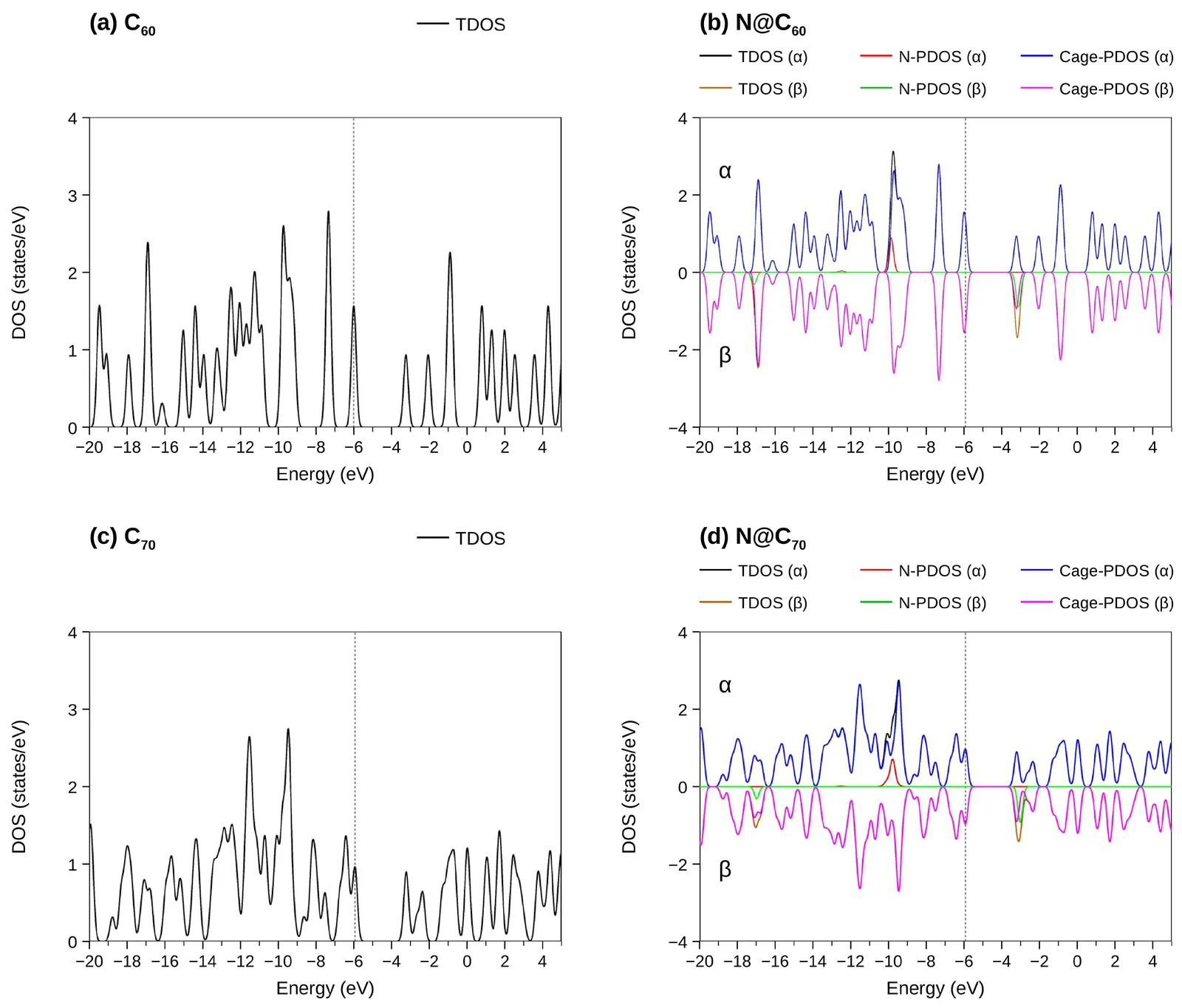
Total densities of states and fragment-projected densities of states of (**a**) C_60_, (**b**) N@C_60_, (**c**) C_70_, and (**d**) N@C_70_. For the open-shell systems, the beta curves are mirrored below zero for visual clarity. The vertical dashed lines mark the HOMO energies; the alpha and beta HOMO energies overlap at the plot resolution.

Figure 3 shows the total density of states for C_60_ and C_70_, together with the separate alpha and beta TDOS and PDOS curves for N@C_60_ and N@C_70_. Since these systems are finite molecules, the curves were obtained by applying Gaussian broadening to discrete molecular orbital energies.

For both endofullerenes, most features in the TDOS are also present in the PDOS of the carbon cage. The carbon framework accounts for most of the electronic states in both spin channels. The alpha and beta cage contributions are also similar across most of the energy range, which suggests that spin polarization on the carbon framework is small.

The nitrogen contribution is limited mainly to a few energy regions and differs between the two spin channels. In the alpha channel, the main nitrogen features occur near −9.9 eV for N@C_60_ and −9.8 eV for N@C_70_. In the beta channel, they occur near −3.1 and −3.0 eV, respectively. The alpha features lie in the occupied region, while the beta features are above the highest occupied molecular orbital energy. This distribution is compatible with the open-shell quartet description of the nitrogen atom.

The occupied nitrogen PDOS features near −9.9 eV for N@C_60_ and −9.8 eV for N@C_70_ lie below their frontier region. For N@C_60_, this ordering is qualitatively consistent with the occupied 2p state derived from nitrogen, whose eigenvalue is −10.413 eV in Greer’s unrestricted B3LYP/TZVP calculation [31]. The broadened PDOS peak positions and the individual orbital eigenvalues are different quantities. Their relative positions nevertheless help explain how spin can be strongly localized on nitrogen while the HOMO and LUMO are located mainly on the cage.

Across most of the spectrum, the contribution from nitrogen is small. Its concentration in a few energy regions suggests that encapsulation does not cause a broad change in the electronic states of the cage. The carbon framework remains the main part of the electronic structure, while nitrogen adds localized contributions that depend on the spin channel. This interpretation agrees with the frontier orbital results and the localization of the spin population on nitrogen discussed above. The interaction between the N atom and the cage is considered further in the NCI and QTAIM analyses.

The small perturbation of the ground state should not be taken to mean complete electronic isolation under all conditions. Laser-flash measurements have shown that the encapsulated nitrogen accelerates excited-state decay in N@C_60_ [57]. This process was not evaluated in the present calculations of the ground state.

### 3.7. Spin Density Distribution

**Figure 4:**
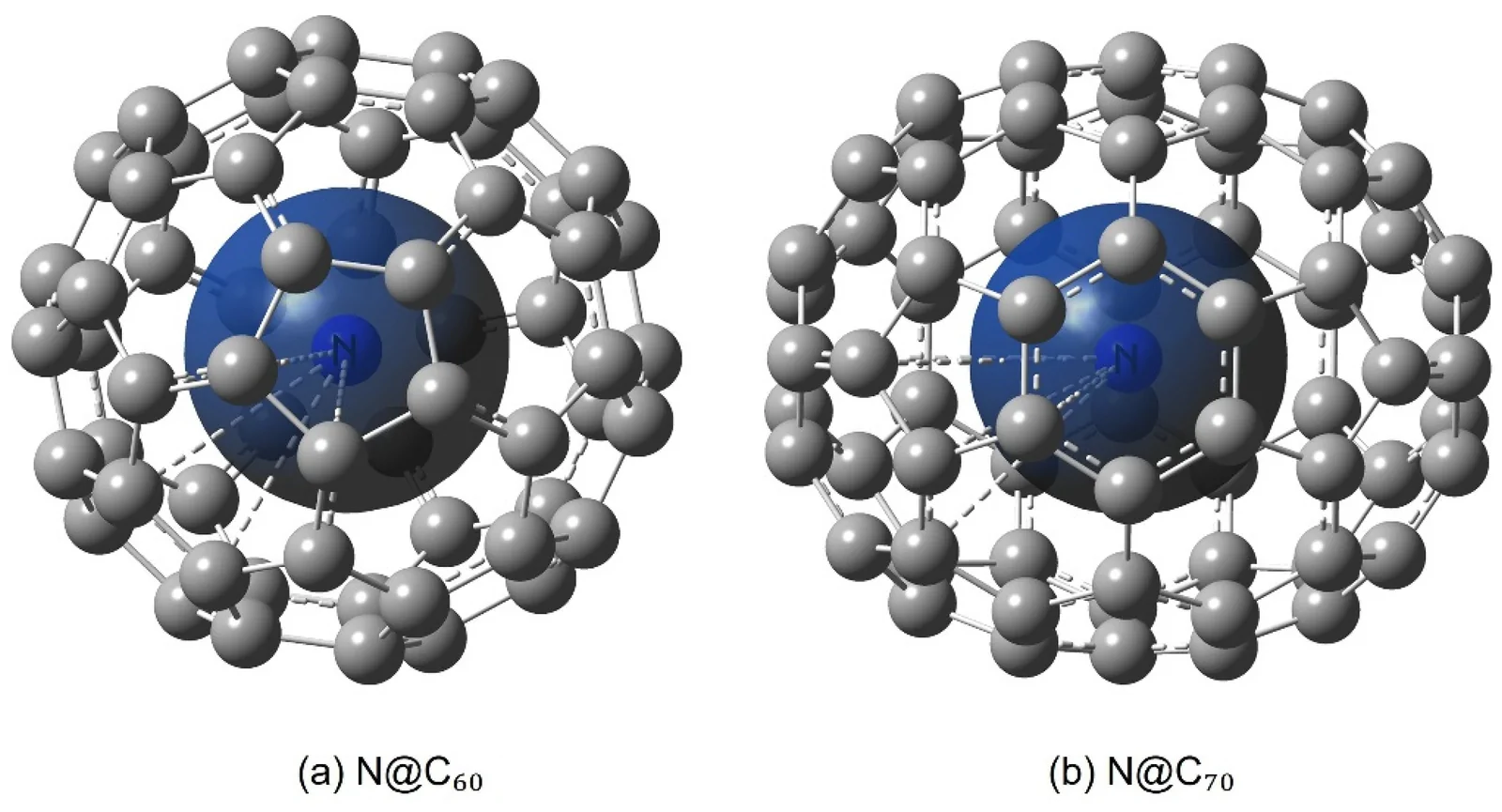
Positive spin-density isosurfaces of (**a**) N@C_60_ and (**b**) N@C_70_ from the UB3LYP/6-31G(d) wavefunctions, plotted at the same isovalue of +4.0 × 10^−4^ a.u. Blue denotes positive *ρ*_α_ − *ρ*_β_. The maps emphasize the spin distribution centered on nitrogen and do not show the negative component. Figure S1 shows positive and negative components separately and together at a lower absolute isovalue.

At the same positive spin-density isovalue of +4.0 × 10^−4^ a.u., the displayed distribution is centered mainly on the encapsulated nitrogen atom in both N@C_60_ and N@C_70_ (Figure 4). The Mulliken and Hirshfeld population analyses assign approximately 94–99% of the net spin population to nitrogen (Table 4). This pattern is consistent with earlier EPR measurements and calculations indicating that nitrogen largely retains its atomic quartet character after encapsulation [10,12,17].

The isosurfaces give a qualitative view and depend on the selected isovalue. Additional maps at ±2.0 × 10^−5^ a.u. show regions with small positive and negative spin densities in each endofullerene (Figure S1). Positive regions extend toward the carbon framework, while negative regions are visible inside the fullerene cavity. Showing the two signs separately reveals features that are partly obscured when both are shown together. Negative spin density means that the local beta electron density exceeds the alpha density; it does not mean that the total electron density is negative. A region without a visible feature at the higher threshold cannot be assumed to have zero spin density. Isosurface extent does not measure an integrated atomic spin population, and a local negative region alone does not establish a negative net spin population on a carbon atom. The maps complement the population analyses for individual atoms and remain consistent, with spin localization predominantly on nitrogen.

### 3.8. Noncovalent Interaction Analysis

**Figure 5:**
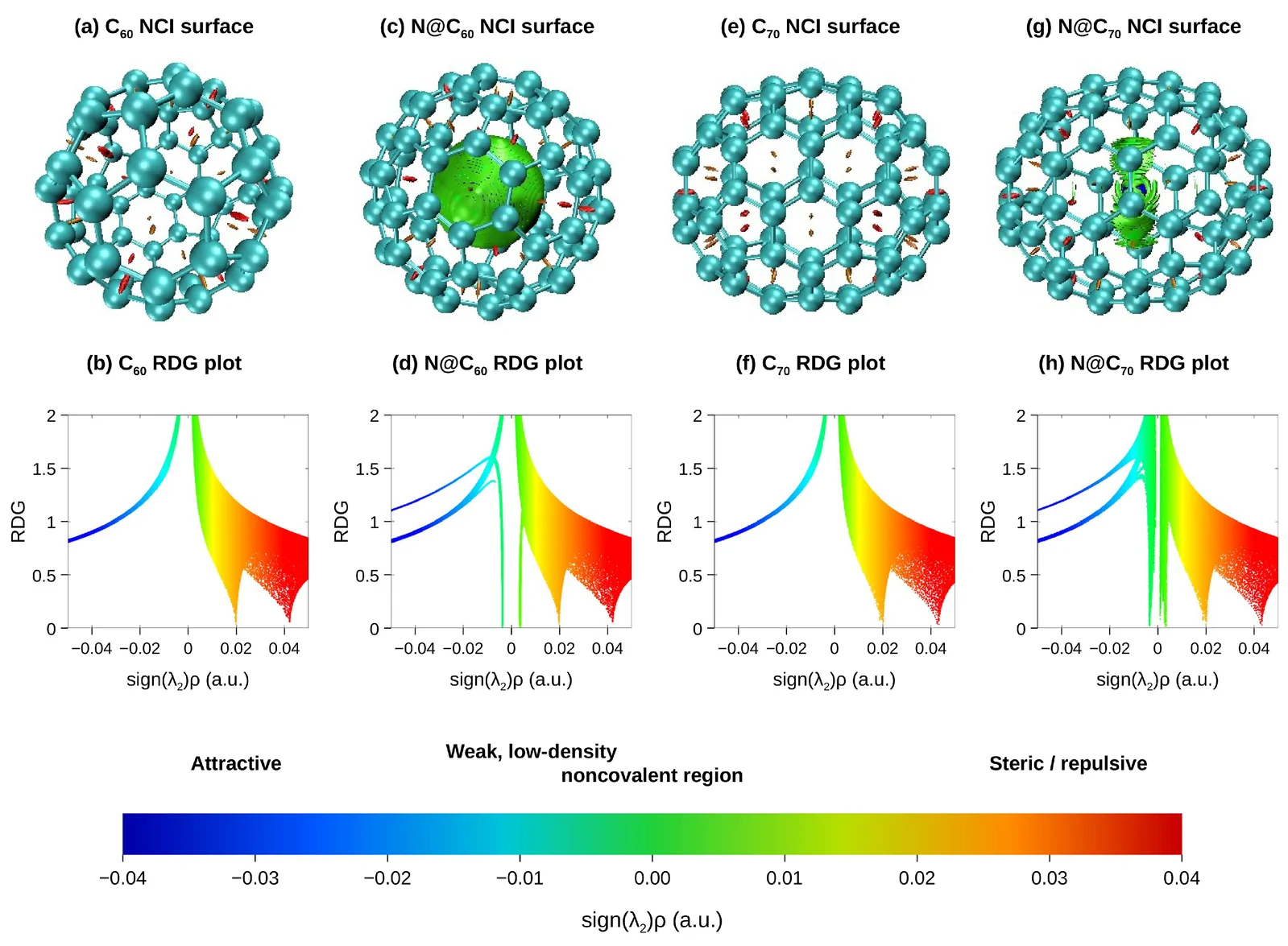
NCI isosurfaces and corresponding plots of RDG against sign(*λ*_2_)*ρ* for (**a**,**b**) C_60_, (**c**,**d**) N@C_60_, (**e**,**f**) C_70_, and (**g**,**h**) N@C_70_. The same color scale was applied to all four systems. The RDG isovalue is 0.5, and the sign(λ_2_)ρ color range is 0.04 to +0.04 a.u.

The empty C_60_ and C_70_ cages were included as reference systems to separate features of the carbon frameworks from those related to nitrogen encapsulation (Figure 5). Their NCI surfaces and RDG plots show the low electron density and steric regions associated with the isolated cages. In N@C_60_ and N@C_70_, additional green surfaces appear between nitrogen and the inner cage wall. The RDG plots also show narrow features near zero on the sign(*λ*_2_)*ρ* axis that are not evident in the empty cages.

In NCI analysis, negative values, values close to zero, and positive values of sign(*λ*_2_)*ρ* are generally associated with attractive interactions, weak contacts in low electron density regions, and steric or repulsive effects, respectively [46]. With the same display settings, the surfaces between nitrogen and the cages are mainly green. A clear blue region is not evident between nitrogen and the carbon framework. These features suggest weak contacts spread over the inner cage surface rather than a strong attractive contact at one location.

The spatial pattern differs between the two endofullerenes. N@C_60_ shows a more even distribution around nitrogen. In N@C_70_, the surfaces are less uniform and follow the elongated shape of the cavity. This difference describes the positions of the contacts. It does not provide a numerical measure of interaction strength on its own.

NCI analysis gives a qualitative view and does not divide the interaction into separate energy terms. Considered together with the counterpoise-corrected energies and the D3(BJ) analysis in Section 3.3, the NCI results are compatible with a weak interaction spread across the cavity. The energy calculations show a stabilizing contribution from the empirical D3(BJ) correction within the adopted model. Surface area and RDG feature intensity were not used as numerical measures. Interaction energies were used for the energetic comparison, while QTAIM was used to examine the electron density topology.

### 3.9. QTAIM Analysis of the N···C Contact Topology

The Poincaré–Hopf relation was used to check the internal consistency of the baseline UB3LYP/6-31G(d) critical point sets:

61 − 150 + 122 − 32 = 1 for N@C_60_

71 − 113 + 50 − 7 = 1 for N@C_70_

**Figure 6:**
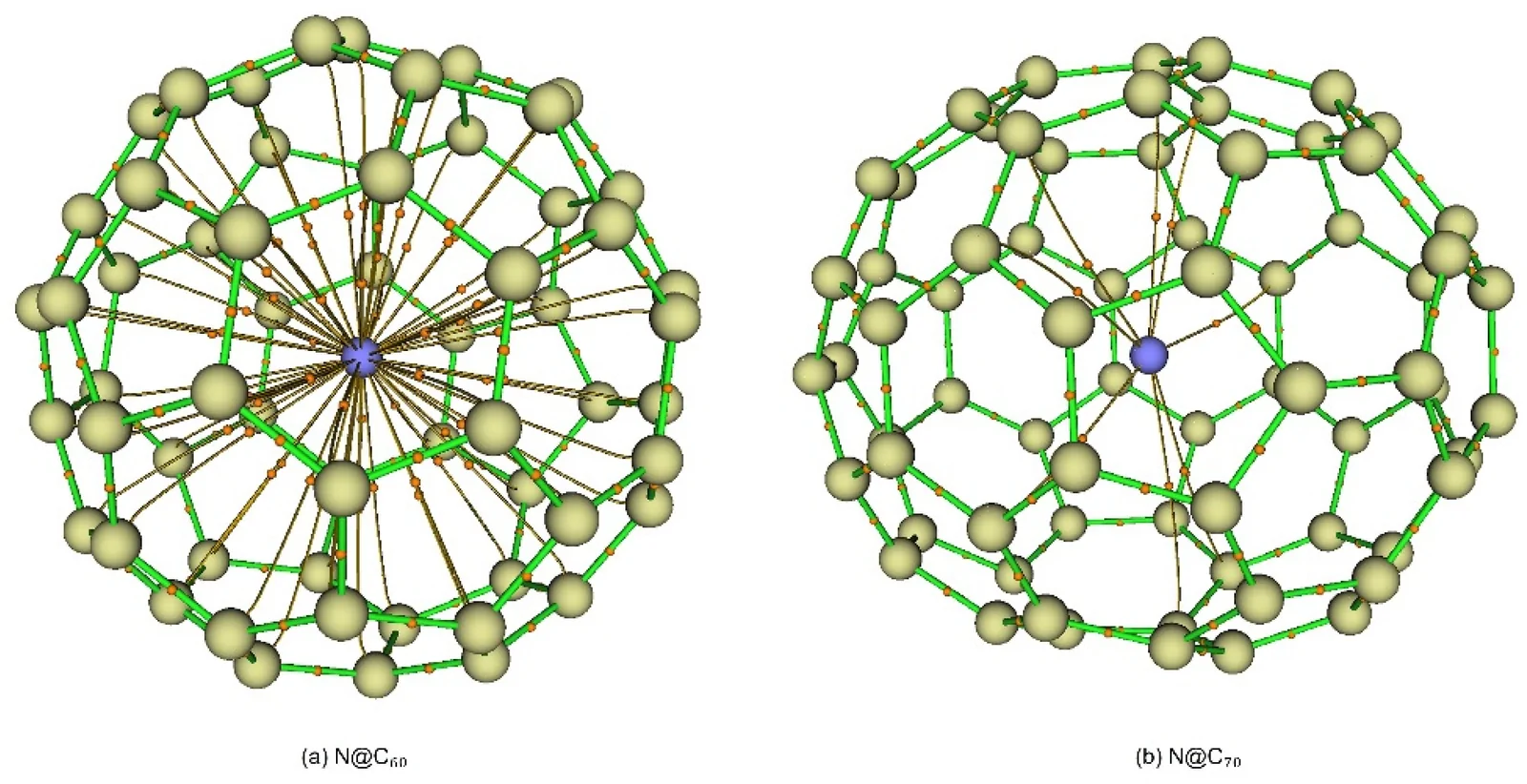
QTAIM molecular graphs of (**a**) N@C_60_ and (**b**) N@C_70_ at the baseline UB3LYP/6-31G(d) level. Orange spheres mark (3, −1) critical points, and brown lines indicate the displayed topological paths.

At the baseline UB3LYP/6-31G(d) level, the QTAIM molecular graph of N@C_60_ contains 60 N···C critical points of type (3, −1) and their associated electron density gradient paths. In the molecular graph, each path extends from nitrogen to a different cage carbon atom. Therefore, all 60 carbon atoms participate in one N···C topological path. The paths are distributed almost uniformly around the nearly central nitrogen atom. This statement refers only to QTAIM connectivity and does not imply the presence of 60 conventional N-C bonds. For N@C_70_, eight N···C critical points and associated paths are found, with an anisotropic distribution in the elongated cavity (Figure 6). The Poincaré–Hopf relation confirms the internal consistency of the topology, but it does not establish the chemical importance or strength of an individual path.

Table 6 summarizes the mean descriptors evaluated at the N···C critical points of the baseline UB3LYP/6-31G(d) densities. The mean *ρ*_b_ values are low, 0.00369 a.u. for N@C_60_ and 0.00348 a.u. for N@C_70_. In both systems, ∇^2^*ρ*_b_ and *H*_b_ are positive, *G*_b_/|*V*_b_| is greater than 1, and ELF is approximately 0.01. These descriptors are consistent with weak closed-shell contacts and little shared electron localization [48,49,50]. The small differences between the two systems do not alter this general classification. The N···C distances in Table 6 refer only to carbon atoms linked to nitrogen by the identified paths and are reported as mean values followed by their ranges. The individual atom pair assignments and critical point descriptors are provided in the S3_QTAIM_Data worksheet of the Supplementary Data workbook. The N@C_70_ baseline means in Table 6 include all eight contacts. They differ from the means over the six matched contacts used for the sensitivity comparison in Table S4.

Tightening the search convergence criteria as described in Section 2.4 did not change the critical point counts or the connected atom pairs for either baseline wavefunction (Table S1). The N@C_60_ and N@C_70_ searches retained 60 and eight N···C bond paths, respectively, with the same carbon atoms assigned to them. The electron density, its Laplacian, G, V, H, and ELF at the critical points were unchanged at the precision printed in the output files. This test examines the search criteria used here; it does not test changes in the electronic structure method.

Table 7 summarizes the comparisons of basis sets and functionals at fixed geometries. N@C_60_ retained the same 60 N···C connections at all three levels. N@C_70_ had eight connections at UB3LYP/6-31G(d), but the same six at UB3LYP/def2-TZVP and UωB97X-D/def2-TZVP. These six connect N71 to C10, C17, C31, C47, C64, and C68; the baseline N71···C65 and N71···C70 contacts were not recovered in either def2-TZVP dataset (Table S5). Atom labels follow the numbering of the optimized N@C_70_ geometry provided in the S1_Coordinates worksheet of the Supplementary Data workbook. The 90 and 105 cage C–C connections remained unchanged for N@C_60_ and N@C_70_, respectively. Full counts of critical points are given in Table S2.

The local descriptors changed on basis enlargement even though N@C_60_ retained the same connectivity (Tables S3 and S4). For UB3LYP, the mean N@C_60_ BCP density decreased by 6.62%, the mean Laplacian increased by 10.12%, and mean H increased from 0.000434667 to 0.000919213 a.u. (+111.48%). For the six matched N@C_70_ contacts, the corresponding changes were −5.03%, +9.20%, and +89.95%. Keeping the same connections therefore does not mean that the local descriptors are unchanged.

At def2-TZVP, replacing UB3LYP with UωB97X-D changed the mean density, Laplacian, and H by −0.16%, +0.47%, and +2.68% for N@C_60_, and by −0.49%, +0.38%, and +1.63% for the six matched N@C_70_ contacts. All identified N···C BCPs retained positive Laplacians and H values and G/|V| > 1. Other descriptors varied more: for example, mean N@C_60_ ellipticity changed from 8.11985 to 14.17313. The results support a contrast between extensive and restricted contact networks across the tested levels; they do not show that the 60 versus eight count is independent of the method. The targeted N@C_70_ search described in the Supplementary Information found only previously identified points. A finite search does not prove that every possible critical point has been located.

A QTAIM path is a feature of the calculated electron density topology and, by itself, does not demonstrate a conventional chemical bond [58]. Accordingly, the path counts should not be interpreted as coordination numbers or as additive measures of the total interaction strength. When considered together with the NCI results and the comparable counterpoise-corrected interaction energies, the QTAIM data support weak N···C contacts in both cages. The principal difference between N@C_60_ and N@C_70_ is therefore the spatial distribution of these contacts rather than their general interaction type.

## 4. Discussion

The calculated structural and spin descriptors are broadly consistent with the available experimental picture of nitrogen endofullerenes. EPR studies indicate that atomic nitrogen remains close to the cage center and largely preserves its ^4^S_3/2_ ground state [10,11,12]. In the present calculations, the nitrogen atom is displaced by only 0.001 Å from the geometric center of C_60_ and by 0.017 Å from that of C_70_, while Mulliken and Hirshfeld analyses assign 94.1–99.3% of the spin population to nitrogen. Available EPR results and earlier calculations also indicate modest differences in the spin environment of N@C_60_ and N@C_70_ [12,13,14,17,31]. In the present work, this similarity in overall spin localization is accompanied by a difference in the QTAIM topology at the baseline UB3LYP/6-31G(d) level: 60 nearly isotropically distributed N···C paths are obtained for N@C_60_, whereas eight anisotropically distributed paths are found for N@C_70_. These path counts describe features of the calculated electron-density topology and should not be interpreted as conventional bond numbers. The selected encapsulation-induced vibrational shifts do not exceed 0.8 cm^−1^, which is compatible with preservation of the established C_60_ and C_70_ cage fingerprints [18,19,20,21,22]. Because hyperfine coupling constants were not calculated and the reported harmonic frequencies are unscaled, these comparisons indicate qualitative consistency with, rather than direct validation by, the experiment.

This comparison describes differences in the confinement environment at the level of individual atoms, while the baseline calculations show that the spin remains predominantly localized on nitrogen. The spin fractions in Table 4 and the separately calculated CP interaction energies in Table 3 are similar for the two cages, but their calculated contact graphs differ markedly. N@C_60_ retains 60 N···C connections at all three density levels tested; N@C_70_ has eight at the baseline level and the same six in both def2-TZVP calculations (Table 7). Similar spin localization and comparable electronic stabilization therefore coexist with different spatial arrangements of the weak contacts. The contact maps show differences that the spin and energy summaries alone do not resolve. Because C_60_ and C_70_ differ in carbon count and cavity dimensions as well as shape, this contrast accompanies the combined change in confinement geometry. Bond paths should not be interpreted as conventional chemical bonds [58]. Their counts are not used here to measure stabilization or spin qubit performance.

The checks separate the effects of numerical search settings from those of the electronic structure method (Section 3.9; Table 7 and Tables S1–S6). Tightening the search criteria left both baseline graphs unchanged. N@C_60_ retained 60 N···C connections across the three levels, while N@C_70_ changed from eight baseline contacts to the same six contacts in both def2-TZVP calculations. The contrast between the extensive and restricted contact networks remains, but the exact N@C_70_ count depends on the computational level. Local descriptors also vary, especially when the basis is enlarged. These checks do not establish convergence to the complete basis set limit, invariance with other functionals or geometries, or greater accuracy than correlated methods. The study does not provide a coupled-cluster or MP2 benchmark, and the QTAIM comparisons do not independently validate the interaction energies.

The interaction energy check concerns a separate quantity. With the geometries and UB3LYP-D3(BJ) settings unchanged, increasing the basis from def2-TZVP to def2-QZVP changes the energies obtained with counterpoise correction by less than 0.05 kcal mol^−1^ for each cage and lowers the BSSE values. This result supports the stability of the energy comparison under the tested basis enlargement. The QTAIM contact counts were examined using separate density calculations, so this energy check does not establish their robustness.

Cavity geometry is a relevant variable in theoretical models of endohedral confinement [59]. The contact maps show how the calculated N···C density connections are arranged around nitrogen and provide a structural reference for later calculations of the spin Hamiltonian. Experimental observations of relaxation differences between cages [15] and spin studies in a structured host [16] make this question relevant to fullerene spin materials [60]. Testing whether specific changes in the contacts are associated with hyperfine anisotropy or zero-field splitting would require those observables to be calculated explicitly. We did not evaluate hyperfine tensors, spin relaxation or coherence times, motion at finite temperature, or effects of the condensed phase.

## 5. Conclusions

This study compares two zero-dimensional carbon nanocages that confine the same atomic nitrogen guest. Encapsulation produces negligible structural reconstruction: the selected vibrational modes shift by no more than 0.8 cm^−1^, the largest HOMO–LUMO gap change is 0.007 eV, and the electronic states remain dominated by the carbon cages. Mulliken and Hirshfeld analyses assign 94–99% of the spin population to nitrogen, while the charge analyses indicate no substantial ionic transfer.

At UB3LYP-D3(BJ)/def2-TZVP, the counterpoise-corrected interaction energies are −9.09 and −8.55 kcal mol^−1^ for N@C_60_ and N@C_70_, respectively. The corresponding def2-QZVP values are −9.13 and −8.52 kcal mol^−1^, with absolute changes of 0.04009 and 0.03302 kcal mol^−1^ from def2-TZVP. These values indicate comparable electronic stabilization at the fixed geometries and little sensitivity to this basis enlargement within the adopted model. At both def2 basis sizes, the empirical D3(BJ) correction changes the positive UB3LYP interaction components into negative total interaction energies. The NCI/QTAIM descriptors are consistent with weak, low-density, closed-shell N···C contacts.

At the fixed geometries, N@C_60_ retains 60 N···C connections at UB3LYP/6-31G(d), UB3LYP/def2-TZVP, and UωB97X-D/def2-TZVP. N@C_70_ has eight contacts at the baseline level and the same six contacts in the two def2-TZVP calculations. The difference between extensive and restricted contact networks is retained across the tested levels, whereas the exact N@C_70_ count and local QTAIM descriptors depend on the computational level. These paths are features of electron-density topology, not conventional bonds or coordination numbers.

In the model of isolated molecules, the change from nearly spherical C_60_ to elongated C_70_ is accompanied by a reorganization of the N···C contact pattern, while the baseline analyses of structure and populations show little change in the host framework and indicate predominantly nitrogen-centered spin. Because both cage size and shape change, their effects are not separated here. Future work could relate the calculated contact topology to hyperfine observables and assess its behavior in condensed-phase environments and at higher levels of electronic-structure theory.

## Figures and Tables

**Table 1:** Structural parameters obtained from the optimized geometries.

Parameter	C_60_	N@C_60_	C_70_	N@C_70_
Mean C–C bond length, Å	1.434	1.434	1.433	1.433
C–C bond length range, Å	1.395–1.453	1.396–1.454	1.389–1.471	1.389–1.471
Mean cage radius, Å	3.550	3.550	3.837	3.837
Cage radius range, Å	3.549–3.550	3.550–3.551	3.563–4.158	3.564–4.157
N displacement from geometric center, Å	—	0.001	—	0.017
Minimum/mean/maximum N···C distance, Å	—	3.549/3.550/3.551	—	3.548/3.837/4.161

**Table 2:** Selected calculated harmonic wavenumbers, dominant motions, IR intensities, and Raman activities.

System	Type	Dominant Motion	ν_empty_	ν_endo_	I/A_empty_	I/A_endo_
C_60_/N@C_60_	IR	Radial cage deformation	538.0	537.4	28.38	28.13
C_60_/N@C_60_	IR	Tangential C–C stretching	1460.3	1459.6	11.81	11.76
C_60_/N@C_60_	Raman	Radial cage breathing	496.8	496.7	129.00	128.32
C_60_/N@C_60_	Raman	High-wavenumber C–C stretching	1503.3	1502.5	409.58	403.52
C_70_/N@C_70_	IR	Low-wavenumber cage deformation	467.8	467.0	11.06	11.02
C_70_/N@C_70_	IR	Cage deformation	544.1	543.9	16.90	16.76
C_70_/N@C_70_	IR	High-wavenumber C–C stretching	1460.7	1461.1	99.77	99.84
C_70_/N@C_70_	Raman	Radial cage breathing	457.6	457.7	144.00	143.32
C_70_/N@C_70_	Raman	High-wavenumber C–C stretching	1480.0	1480.5	262.92	241.91

Wavenumbers are given in cm^−1^. For IR modes, I/A denotes IR intensity in km mol^−1^; for Raman modes, it denotes Raman activity in Å^4^ amu^−1^. Each assignment describes the dominant carbon cage motion observed in the calculated normal mode animation and does not imply a pure normal coordinate. The subscripts “empty” and “endo” refer to the empty cage and the corresponding nitrogen endofullerene, respectively.

**Table 3:** Counterpoise-corrected interaction energies and BSSE values (kcal mol^−1^).

Level of Theory	ΔE_int,CP_ N@C_60_	ΔE_int,CP_ N@C_70_	BSSE N@C_60_	BSSE N@C_70_
UB3LYP/6-31G(d)	+3.77	+2.52	2.56	2.26
UB3LYP-D3(BJ)/def2-TZVP	−9.09	−8.55	0.48	0.41
UB3LYP-D3(BJ)/def2-QZVP	−9.13	−8.52	0.12	0.16

**Table 4:** Nitrogen atomic charges and spin populations.

Descriptor	N@C_60_	N@C_70_
Mulliken charge on N, e	−0.001	−0.004
Hirshfeld charge on N, e	+0.111	+0.083
CM5 charge on N, e	+0.092	+0.071
Mulliken spin population on N	2.972	2.978
Mulliken spin fraction on N, %	99.1	99.3
Hirshfeld spin population on N	2.823	2.865
Hirshfeld spin fraction on N, %	94.1	95.5

**Table 5:** Frontier orbital energies and HOMO–LUMO gaps (eV).

System	Spin Channel	E_HOMO_	E_LUMO_	ΔE
C_60_	Closed-shell	−5.986	−3.226	2.761
N@C_60_	Alpha	−5.986	−3.225	2.761
N@C_60_	Beta	−5.984	−3.230	2.754
C_70_	Closed-shell	−5.922	−3.236	2.685
N@C_70_	Alpha	−5.920	−3.234	2.686
N@C_70_	Beta	−5.919	−3.233	2.686

**Table 6:** Mean QTAIM and related real-space descriptors at the N···C (3, −1) critical points at the baseline UB3LYP/6-31G(d) level.

Descriptor	N@C_60_	N@C_70_
Number of N···C (3, −1) critical points	60	8
Mean N···C distance (range) for carbon atoms linked by the identified paths, Å	3.550 (3.549–3.551)	3.562 (3.548–3.581)
ρ_b_, a.u.	0.00369	0.00348
∇^2^ρ_b_, a.u.	+0.01333	+0.01195
*H*_b_, a.u.	+0.000435	+0.000437
*G*_b_/|*V*_b_|	1.18	1.21
ELF at the critical point	0.00941	0.01018

**Table 7:** N···C bond critical points and associated atom-pair connections found at the fixed geometries across three computational levels.

Computational Level	N@C_60_	N@C_70_
UB3LYP/6-31G(d)	60	8
UB3LYP/def2-TZVP	60	6
UωB97X-D/def2-TZVP	60	6

## Data Availability

The data presented in this study are available in the article, the Supplementary Information document, the original Supplementary Data workbook, and the QTAIM Sensitivity Data workbook. Additional calculation files are available from the corresponding author upon reasonable request.

## Supplementary Materials

The following supporting information can be downloaded at https://www.mdpi.com/article/10.3390/nano16211349/s1, the original Supplementary Data workbook (baseline data); Supplementary Information, which contains Figure S1 (separate and combined views of positive and negative spin density) and Tables S1–S6 (checks of search convergence, comparisons of basis sets and functionals, atom pair assignments, and details of the electronic calculations); and the QTAIM Sensitivity Data workbook, which contains comparison records for individual atom pairs and summaries based on formulas.

## Abbreviations

The following abbreviations are used in this manuscript:

DFT	Density functional theory
EPR	Electron paramagnetic resonance
B3LYP	Becke three-parameter Lee–Yang–Parr functional
UB3LYP	Unrestricted B3LYP
D3(BJ)	Grimme D3 dispersion correction with Becke–Johnson damping
SCF	Self-consistent field
HOMO	Highest occupied molecular orbital
LUMO	Lowest unoccupied molecular orbital
TDOS	Total density of states
PDOS	Projected density of states
BSSE	Basis set superposition error
NCI	Noncovalent interaction
RDG	Reduced density gradient
QTAIM	Quantum theory of atoms in molecules
CM5	Charge model 5
RMSD	Root-mean-square deviation
VMD	Visual Molecular Dynamics
UωB97X-D	Unrestricted ωB97X-D functional
