# Supplementary material for: Cage Geometry Reorganizes N···C Contacts While Preserving Nitrogen Spin Localization in N@C60 and N@C70: A DFT Study
Source: Nanomaterials (Basel). 2026 Oct 24;16(21):1349. doi: 10.3390/nano16211349 (PMC13764878; doi:10.3390/nano16211349)
Supplement: Supplementary file 1 [file nanomaterials-16-01349-s001.zip › Supplementary Information.pdf]

Supplementary Information_nanomaterials_4632437

Figure S1

Positive and negative spin-density views at the same absolute isovalue

(a) N@C_{60} — Positive only

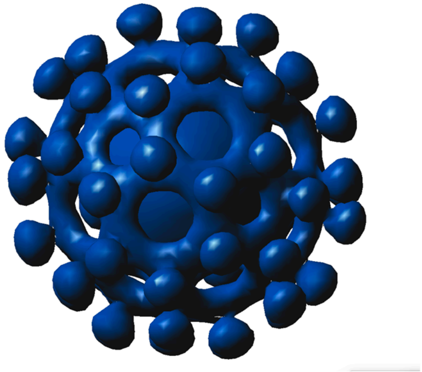

(b) N@C_{60} — Negative only

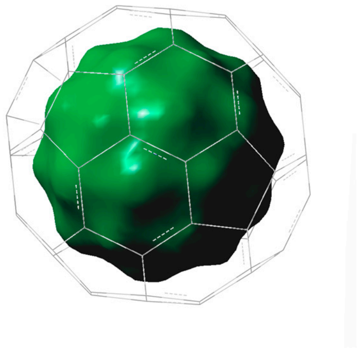

(c) N@C_{60} — Both signs

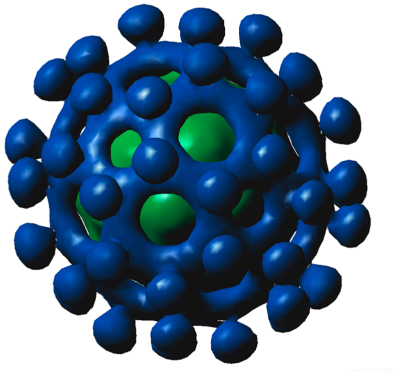

(d) N@C_{70} — Positive only

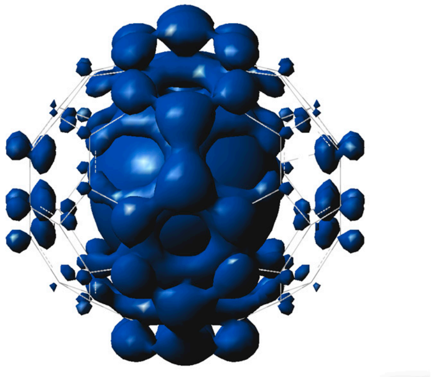

(e) N@C_{70} — Negative only

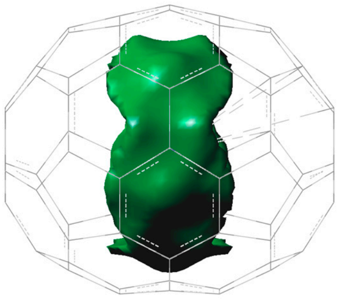

(f) N@C_{70} — Both signs

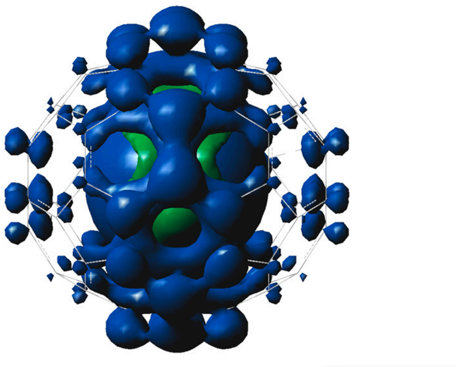

Figure S1. Spin-density isosurfaces of N@C_{60} (a–c) and N@C_{70} (d–f) from the UB3LYP/6-31G(d) SCF densities. Positive-only (a,d), negative-only (b,e), and combined-sign (c,f) views are shown at $\rho_s = +2.0 \times 10^{-5}$ a.u. (blue) and $\rho_s = -2.0 \times 10^{-5}$ a.u. (green), where $\rho_s(r) = \rho_\alpha(r) - \rho_\beta(r)$. The carbon cages are shown as wireframes. These lower-threshold views display low-magnitude regions of both signs and complement Figure 4. Surface extent should not be interpreted as an atomic spin population.

Table S1. Sensitivity to CP-search convergence criteria

The UB3LYP/6-31G(d) wavefunction and optimized geometry of each endofullerene were held fixed. Multiwfn 2026.1.12 was used for both searches; the tighter search was started from scratch.

Search setting	Baseline	Tighter
Maximum iterations	120	200
Gradient-norm threshold (a.u.)	10^{-6}	10^{-7}
Displacement threshold (bohr)	10^{-7}	10^{-8}

System / search	(3,−3)	(3,−1)	(3,+1)	(3,+3)	Total	N···C	C–C	Path branches
N@C_{60} / Baseline	61	150	122	32	365	60	90	300
N@C_{60} / Tighter	61	150	122	32	365	60	90	300
N@C_{70} / Baseline	71	113	50	7	241	8	105	226
N@C_{70} / Tighter	71	113	50	7	241	8	105	226

The Poincaré–Hopf sums are 61 − 150 + 122 − 32 = 1 for N@C_{60} and 71 − 113 + 50 − 7 = 1 for N@C_{70}, under both settings. These are consistency checks, not proof of exhaustive critical-point recovery.

The N···C column counts bond critical points and their associated full bond paths, not individual path branches. Each bond critical point has two branches. Nitrogen is connected to C1–C60 in N@C_{60}; in N@C_{70}, N71 is connected to C10, C17, C31, C47, C64, C65, C68, and C70 under both settings.

Unchanged settings: Newton algorithm; step scale 1.0; minimum CP separation 0.03 bohr; atomic-distance screening factor 1.50; Hessian singularity threshold 5×10^{-22}; all CP types retained. Starting points were nuclear positions, pair midpoints, triangle centers, and tetrahedron centers. Path settings: 0.03 bohr step, 451 maximum points, and 0.05 bohr stopping distance.

Across all critical points, ρ, $\nabla^2\rho$, G, V, H, and ELF were identical at the printed precision in each baseline/tighter pair. Small changes in gradient-related quantities did not alter the classifications or connectivity. This test does not vary the wavefunction, path step, CP-merging distance, or NCI grid.

Table S2. Critical-point counts and contact connectivity

All comparisons use the fixed UB3LYP/6-31G(d) optimized nuclear geometry for each system. Level A: UB3LYP/6-31G(d); level B: UB3LYP/def2-TZVP; level C: UωB97X-D/def2-TZVP. The tighter search protocol in Table S1 was used for these comparisons.

System / level	(3,−3)	(3,−1)	(3,+1)	(3,+3)	Total	N···C	C–C	Path branches
N@C_{60} / A	61	150	122	32	365	60	90	300
N@C_{60} / B	61	150	122	32	365	60	90	300
N@C_{60} / C	61	150	122	32	365	60	90	300
N@C_{70} / A	71	113	50	7	241	8	105	226
N@C_{70} / B	71	111	48	7	237	6	105	222
N@C_{70} / C	71	111	48	7	237	6	105	222

The Poincaré–Hopf sum is 1 for all six datasets. Each bond critical point has two branches ending at its assigned nuclear critical points. This checks the exported graph and labels; it is not an independent reintegration of the paths or proof that every critical point was recovered.

The same 60 N···C and 90 cage C–C atom pairs occur in all three N@C_{60} datasets. The 105 cage C–C pairs also agree in all three N@C_{70} datasets. Both def2-TZVP N@C_{70} densities yield the same six N···C pairs (Table S5). CP indices change between calculations and were not used as identifiers for the comparisons.

Table S3. $N@C_{60}$: matched N···C BCP descriptors

Arithmetic means over the same 60 atom pairs at levels A, B, and C (defined in Table S2). For Tables S3 and S4, ΔB/A measures the UB3LYP basis-set change and ΔC/B measures the functional change at def2-TZVP. Percentages compare means, not uncertainties.

Descriptor	A mean	B mean	C mean	ΔB/A	ΔC/B
ρ	0.003686661	0.003442456	0.003436783	-6.62%	-0.16%
$\nabla^2\rho$	0.013331838	0.014680434	0.014749944	+10.12%	+0.47%
G	0.002898293	0.002750895	0.002743639	-5.09%	-0.26%
|V|	0.002463626	0.001831682	0.001799793	-25.65%	-1.74%
H	0.000434667	0.000919213	0.000943847	+111.48%	+2.68%
G/|V|	1.176434	1.501842	1.524420	+27.66%	+1.50%
ELF	0.009414235	0.008558838	0.008261139	-9.09%	-3.48%
Spin density	0.001027824	0.000990477	0.000917609	-3.63%	-7.36%
Ellipticity	4.231478	8.119853	14.173128	+91.89%	+74.55%

Units for Tables S3 and S4: ρ and spin density, electrons $bohr^{-3}$; $\nabla^2\rho$, electrons $bohr^{-5}$; G, V, and H, hartree $bohr^{-3}$. G/|V|, ELF, and ellipticity are dimensionless. V is negative for every listed contact; its magnitude is tabulated. G/|V| is averaged over the individual ratios.

Selected ranges over the matched contacts

Descriptor range	A	B	C
ρ	0.00367871–0.00369599	0.00343456–0.00345109	0.00342867–0.00344590
$\nabla^2\rho$	0.01330806–0.01336064	0.01464877–0.01471899	0.01471762–0.01479123
H	0.00043414–0.00043531	0.00091763–0.00092110	0.00094222–0.00094589
G/|V|	1.176219–1.176621	1.501242–1.502526	1.523565–1.525207
Ellipticity	3.650860–4.922752	6.393583–10.625095	9.217233–25.963510

All 60 contacts connect N61 to C1–C60. The mean H changes by more than a factor of two on basis enlargement. The large changes in ellipticity also show that preservation of the contact graph does not imply invariance of all local descriptors.

All matched contacts retain $\nabla^2\rho > 0$, $H > 0$, and $G/|V| > 1$ at the three levels.

Table S4. N@C_{70}: matched N···C BCP descriptors

Arithmetic means over the same 6 atom pairs at levels A, B, and C (defined in Table S2). Units, averaging conventions, and percentage definitions are given with Table S3.

Descriptor	A mean	B mean	C mean	ΔB/A	ΔC/B
ρ	0.003460617	0.003286659	0.003270584	-5.03%	-0.49%
$\nabla^2\rho$	0.011874857	0.012967882	0.013017634	+9.20%	+0.38%
G	0.002533470	0.002415208	0.002414199	-4.67%	-0.04%
|V|	0.002098226	0.001588445	0.001573989	-24.30%	-0.91%
H	0.000435244	0.000826763	0.000840210	+89.95%	+1.63%
G/|V|	1.207501	1.520711	1.534068	+25.94%	+0.88%
ELF	0.010080081	0.009544746	0.009127583	-5.31%	-4.37%
Spin density	0.000952679	0.000913692	0.000849316	-4.09%	-7.05%
Ellipticity	3.217294	9.180468	8.559072	+185.35%	-6.77%

Selected ranges over the matched contacts

Descriptor range	A	B	C
ρ	0.00335739–0.00357487	0.00319084–0.00339505	0.00317278–0.00338059
$\nabla^2\rho$	0.01158569–0.01219567	0.01259714–0.01338411	0.01263710–0.01344321
H	0.00042964–0.00044219	0.00080978–0.00084628	0.00082278–0.00086002
G/|V|	1.204291–1.210910	1.511820–1.529363	1.524156–1.543563
Ellipticity	2.242967–4.602140	3.765582–18.704516	3.676801–17.068512

The shared set is N71···C10, C17, C31, C47, C64, and C68. The baseline N71···C65 and N71···C70 contacts are excluded only from these matched means. A missing contact is not represented by zero. Table 6 of the main text retains the means over all eight baseline N@C_{70} contacts.

All matched contacts retain $\nabla^2\rho > 0$, $H > 0$, and $G/|V| > 1$ at the three levels.

Table S5. $N@C_{70}$ atom-pair assignments across the three levels

Nitrogen is N71 throughout. Numbers are bond critical-point indices, not atom indices. Levels A, B, and C are defined in Table S2.

Atom pair	A: B3LYP/6-31G(d)	B: B3LYP/TZVP	C: ωB97X-D/TZVP
N71···C10	189	182	183
N71···C17	198	193	194
N71···C31	178	175	176
N71···C47	126	125	125
N71···C64	114	112	112
N71···C65	109	Not recovered	Not recovered
N71···C68	147	146	145
N71···C70	159	Not recovered	Not recovered

Both def2-TZVP calculations give the same six carbon assignments. The two additional baseline connections were not recovered by these finite searches; this does not prove their absence. The Raw_NC worksheet of the QTAIM Sensitivity Data workbook contains all available contact descriptors, including the two baseline-only contacts.

Full $N@C_{60}$ assignments are provided in the same workbook. The graph remains connected to all 60 cage carbons at each level. CP indices and coordinates can differ even when the atom-pair connection is unchanged.

Table S6. Electronic calculation details and targeted-search record

Four additional fixed-geometry density calculations used Gaussian 16 C.01 with an ultrafine grid, tight SCF convergence, NoSymm, and Stable=Opt. In each case the stability calculation and following checkpoint-based single-point stage terminated normally; the wavefunction was stable under the perturbations tested.

Job	System	Functional	NBasis / NBsUse	SCF energy (hartree)	⟨S^2⟩
03	N@C_{60}	UB3LYP	1891 / 1891	−2341.47871052	3.7536
04	N@C_{70}	UB3LYP	2201 / 2200	−2722.72731839	3.7537
05	N@C_{60}	UωB97X-D	1891 / 1891	−2340.71873355	3.7570
06	N@C_{70}	UωB97X-D	2201 / 2200	−2721.82871040	3.7569

All four use def2-TZVP and neutral quartet states. ⟨S^2⟩ values are before spin-contaminant annihilation. NBasis is the defined basis-function count and NBsUse is the retained count reported by Gaussian. The N@C_{70} values are 2201 and 2200, respectively. These total SCF energies are not N–cage interaction energies.

Limited targeted search for N@C_{70} / UB3LYP / def2-TZVP (job 04)

A separate search used 76 starting coordinates in bohr around the two baseline-only N···C BCPs and their nearby ring critical points. It returned 11 distinct critical points (three bond, five ring, and three cage points). All 11 were already present in the 237-point job-04 dataset. This incomplete local subset had no nuclear critical points and a Poincaré–Hopf sum of −1; it was not added to the full 237-point molecular graph or its count.
